# Nonalcoholic Fatty Liver Disease and Coronary Artery Disease: Big Brothers in Patients with Acute Coronary Syndrome

**DOI:** 10.1155/2020/8489238

**Published:** 2020-04-13

**Authors:** Mauricio Montemezzo, Ahmed AlTurki, Fabio Stahlschmidt, Marcia Olandoski, Jean Rodrigo Tafarel, Dalton Bertolin Precoma

**Affiliations:** ^1^Department of Internal Medicine, Pontifícia Universidade Católica do Paraná, Curitiba, Paraná, Brazil; ^2^Division of Cardiology, McGill University Health Center, Montreal, Canada

## Abstract

**Background:**

The prevalence of nonalcoholic fatty liver disease (NAFLD) has been increasing. This study aimed to evaluate the prevalence of NAFLD, as diagnosed by ultrasound, in patients with acute coronary syndrome (ACS) and to assess whether NAFLD is associated with the severity of coronary obstruction as diagnosed by coronary angiography.

**Methods:**

We performed a prospective single-center study in patients hospitalized due to acute coronary syndrome who underwent diagnostic coronary angiography. Consecutive patients who presented to the emergency room were diagnosed with acute coronary syndrome and were included. All patients underwent ultrasonography of the upper abdomen to determine the presence or absence of NAFLD; NAFLD severity was graded from 0 to 3 based on a previously validated scale. All patients underwent diagnostic coronary angiography in the same hospital, with the same team of interventional cardiologists, who were blinded to the patients' clinical and ultrasonographic data. CAD was then angiographically graded from none to severe based on well-established angiographic criteria.

**Results:**

This study included 139 patients, of whom 83 (59.7%) were male, with a mean age of 59.7 years. Of the included patients, 107 (77%) patients had CAD, 63 (45%) with serious injury. Regarding the presence of NAFLD, 76 (55.2%) had NAFLD including 18 (23.6%) with grade III disease. In severe CAD, 47 (60.5%) are associated with NAFLD, and 15 (83.3%) of the patients had severe CAD and NAFLD grade III.

**Conclusions:**

NAFLD is common in patients with ACS. The intensity of NAFLD detected by ultrasonography is strongly associated with the severity of coronary artery obstruction on angiography.

## 1. Introduction

Nonalcoholic fatty liver disease (NAFLD) is an important public health problem worldwide and is considered one of the most common form of chronic liver disease in the western world [1, 2]. This condition affects approximately 15–30% of the general population, and its prevalence increases in people with metabolic syndrome [3, 4]. The explanation for the high prevalence of NAFLD is the obesity epidemic [2]. Insulin resistance and adiposity are associated with increased lipid influx into the liver and increased de novo hepatic lipogenesis which promote hepatic triglyceride accumulation [2, 4]. NAFLD is considered a hepatic manifestation of metabolic syndrome [2].

The association of NAFLD and metabolic syndrome brings the interest of the possible correlation between NAFLD and cardiovascular artery disease (CAD) [2]. In contrast, studies show that NAFLD is not only associated with CAD in patients who have classical risk factors, but is also correlated with cardiovascular events independent of age, sex, LDL cholesterol, smoking, and features of metabolic syndrome [2]. In addition, some authors have been demonstrating a marked increase in carotid artery intima-media thickness, as an index of subclinical atherosclerosis, in patients with NAFLD [3].

Cardiovascular disease accounts for >30% of deaths in the United States and is the leading cause of mortality in patients with NAFLD, highlighting the importance of recognizing and addressing CAD in patients with NAFLD [5]. In patients with acute myocardial infarction, the prevalence of NAFLD is higher compared to general population and predicts its severity and extent [6].

The main aim of this study was to evaluate the prevalence of NAFLD (diagnosed by ultrasound) in patients with acute coronary syndrome (ACS) and to establish whether NAFLD is associated with the severity of coronary obstruction diagnosed by coronary angiography.

## 2. Materials and Methods

This was a prospective study, between March 2015 and March 2016, performed at the Cardiology department of Hospital Santa Casa de Curitiba (quaternary care medical Centre), from Pontifícia Universidade Católica do Paraná (PUCPR), in Brazil. All study participants gave written informed consent. The study protocol conformed to the ethical guidelines of the 1975 Declaration of Helsinki as reflected in a priori approval by the Ethics Committee in Research at the PUCPR.

All adult patients (≥18 years of age) who presented to the emergency room with ACS were eligible for inclusion. Patients with a history of ≥10 g of daily alcohol consumption, pregnant women, terminal disease, known history of liver disease, any acute illness other than acute coronary syndrome, use of statins, estrogens, steroids, amiodarone, or chemotherapeutic agents, and patients with known CAD, documented in a previous coronary angiography, or with a history of percutaneous coronary intervention or surgical revascularization were excluded from the study.

Diagnosis of ACS was made by typical ongoing ischemic chest pain for >30 minutes and submitting all patients to 12-lead electrocardiogram and serial quantitation of creatine kinase (CK), creatine kinase-MB isoform (CK-MB), and troponin I levels at admission and 3 hours and 6 hours after admission. The results categorized the ACS as follows:

- Acute myocardial infarction with ST-segment elevation (STEMI): ST elevation ≥1 mm in ≥2 contiguous leads (2 mm for leads V1 to V3).
- Acute myocardial infarction without ST-segment elevation (NSTEMI): patients who did not meet the electrocardiographic criteria for STEMI and who had elevated enzymes of myocardial injury.
- Unstable angina (UA): patients who did not meet the criteria for STEMI and NSTEMI, but had more than three cardiovascular risk factors and typical thoracic pain.

All patients underwent diagnostic coronary angiography in the same hospital, with the same team of interventional cardiologists, who were blinded to the patients' clinical and ultrasonographic data. A standard cardiac catheterization procedure with selective contrast injections in the right and left coronary artery system was performed. The data of coronary angiography were evaluated by the angiographic projection in which the coronary lesion was more significant [7]. CAD was classified as follows:

- No apparent CAD: absence of obstruction;
- Mild: obstructive impairment of 0% to 50% of the circumference of the vessel;
- Moderate: obstructive impairment of 51% to 70% of the circumference of the vessel;
- Severe: obstructive impairment greater than or equal to 71% of the circumference of the vessel.

Although liver biopsy is the gold standard test to diagnose NAFLD, it was not performed due to the invasive nature of the test and ethical concerns. To investigate the presence of NAFLD, patients underwent upper abdominal ultrasound (at the bedside), with the same equipment (Sonosite M-Turbo model 2009-2012), during the first 12 hours of the coronary angiography, by an experienced radiologist, who was blinded to participants' details (clinical data and coronary angiography findings). Hepatic steatosis was diagnosed on the basis of characteristic sonographic features, i.e., evidence of diffuse hyperechogenicity of the liver relative to kidneys, ultrasound beam attenuation, and poor visualization of intrahepatic vessel borders and the diaphragm. The severity of hepatic steatosis was assigned using the classification of Hamaguchi and Saadeh [8, 9]:

- Grade 0: absence of hepatic steatosis.
- Grade I: minimal increase in hepatic echogenicity with normal visualization of the diaphragm and the edges of intrahepatic vessels.
- Grade II: mild increase in hepatic echogenicity and slightly altered view of the intrahepatic vessels and diaphragm.
- Grade III: insufficient visualization of the posterior segment of the right lobe and visualization rather poor or zero of the hepatic vessels and diaphragm.

Data on age, sex, clinical data (hypertension and type two diabetes), and detailed information on smoking status, daily alcohol consumption, and use of medications were obtained from all patients. Serum alanine aminotransferase (ALT), aspartate aminotransferase (AST), gamma-glutamyltransferase (GGT), alkaline phosphatase (AP), INR, creatinine, total cholesterol, LDL cholesterol, HDL cholesterol, and triglycerides were also obtained.

The results of quantitative variables were described as mean, median, minimum and maximum values, and standard deviations. Qualitative variables were described as frequencies and percentages. Regarding quantitative variables, to compare the two groups, was considered the Student's *t*-test for independent samples or the nonparametric Mann–Whitney test, when appropriate. For evaluation of the qualitative variables was used Fisher's exact test or the chi-square test. The condition of normality of the variables was evaluated by the Kolmogorov–Smirnov test. For the multivariate analysis, a logistic regression model was adjusted, considering the Wald test for assessing the significance of the explanatory variables. A *P* value of <0.05 was considered statistically significant. Statistical analyzes were performed by SPSS version 20.0 (SPSS Inc., Chicago, IL).

## 3. Results

The cohort study included 139 consecutive patients with ACS, with a mean age of 58.1 ± 12.1 years and 83 were (59.7%) male. Based on ACS characteristics, there were 40 (29.1%) STEMI, 51 (36.6%) NSTEMI, and 48 (34.3%) UA. All patients were in Killip I-II class. Systemic arterial hypertension was present in 71 patients (51.1%) and type two diabetes mellitus in 45 (32.4%). Patient characteristics are summarized in Table 1.

Coronary angiography demonstrated CAD in 107 patients (76, 9%): 10 mild, 34 moderate, and 63 with severe obstruction. Upper abdominal ultrasound showed NAFLD in 76 cases (55.1%): grade I in 36, grade II in 23, and grade III in 18 patients. The correlation between the presence of NAFLD and CAD is represented in Table 2 and its severity, in Table 1. There is association between the presence of NAFLD and severity of CAD (*P* < 0.001). The severity of NAFLD has correlation with coronary angiography severity (*P* < 0.001). Post hoc sample size calculations showed a required sample size of 26 for 90% power and an alpha of 0.01; the post hoc power is 100%.

## 4. Discussion

For many years, the presence of NAFLD was considered as a benign manifestation with no clinical significance [3] but is now considered one of the most important risk factors for cirrhosis and hepatocellular carcinoma [1, 10]. Increasing recognition of the importance of NAFLD and its strong relationship with insulin resistance and metabolic syndrome has stimulated an interest in the association of NAFLD and cardiovascular disease (CVD) and cardiovascular events (CVE) [11–13].

The risk of CVE in subjects with NAFLD was higher than those without NAFLD [5, 11]. There is ample evidence suggesting that NAFLD is linked to an increased coronary artery calcification and high CVD morbidity and mortality, independent of traditional risk factors and components of the metabolic syndrome [7, 8, 11, 14]. NAFLD patients have a decreased survival as compared with general population, and CVD is the leading cause of death, especially in NASH population [11]. Advanced liver fibrosis has been shown to be associated with increasing atherosclerosis by inducing endothelial dysfunction which is independent of cardiovascular risk factors [15].

The exact mechanisms for this complex relationship are not clear [1]. There is growing evidence to suggest that NAFLD is not merely a marker of CVD, but may also be involved in its pathogenesis, possibly through the systemic release of inflammatory and procoagulant factors from the steatotic/inflamed liver [9, 11]. Inflammation is associated with peripheral insulin resistance resulting in increased lipolysis in adipose tissue and increased liver synthesis of triglycerides [1, 11]. As a consequence of abnormal fat accumulation in the hepatocytes, there is a marked derangement in the insulin signaling pathways in the liver [1].

The systemic inflammation state in NAFLD patients could further enhance the presence of atherogenic dyslipidemia (an increase in LDL-c, TG, and apolipoprotein B and decrease in HDL) and increased carotid intima-media thickness, resulting in more CVE in these individuals [1, 5, 11]. The histological severity of NAFLD and inflammation is strongly associated with increased risk of CVD and this atherogenic lipid profile [1]. This suggests that NAFLD may be a marker and an early mediator of atherosclerosis [16].

Analyzing all this data easily supports a role of NAFLD in facilitating the development and progression of CAD in terms of angiographic appearance [17]. There is evidence that presence of NAFLD causes more severe coronary artery disease, and patients with NAFLD are known to have more complex coronary artery disease in angiography [10]. On the other hand, the functional ischemic significance of NAFLD-related coronary lesions, especially in patients with acute coronary syndromes, is not completely studied [17].

Agaç et al. studied 80 patients admitted with ACS and found that the presence of NAFLD is associated with higher SYNTAX Score (SS) [6]. The SS is an angiographic scoring system relating to CAD complexity that is derived from the coronary anatomy and lesion characteristics [6]. These authors found NAFLD as the one of the 2 predictors of supramedian SS in a multivariate model with an OR of 13.20 (95% CI, 2.52–69.15) [6]. Ling and Shu-zheng showed that patients with NAFLD had a high severity of CAD [16]. In their study, NAFLD was present in 45.7% of the 542 patients suspected to have CAD [16]. Boddi et al. showed that severe NAFLD independently increased the risk for multivessel CAD associated to CV events in nondiabetic patients admitted with STEMI [17]. Emre et al. documented that patients with moderate-to-severe NAFLD are more likely to have impaired myocardial perfusion which may contribute to more in hospital major adverse cardiac events and new-onset heart failure [9]. Perera et al. showed in their study a high mortality predicted by the GRACE score among NAFLD patients (adjusted OR of 31 and 15.6) [10]. This study shows that age and NAFLD are important factors contributing to cardiovascular disease-related deaths [10]. In the study by Agaç et al., the prevalence of NAFLD was 81.2% [6]. In the Boddi et al. study, the overall prevalence of NAFLD was 87% [17]. We too included patients in the whole spectrum of ACS. In contrast, Perera et al. found a NAFLD prevalence of 46.7% among their study participants with ACS [10]. The higher proportion of NAFLD in those studies could be due to the increased prevalence of obesity. One significant challenge for mass screening for NAFLD is the lack of a sensitive and specific biochemical marker [1].

### 4.1. Study Limitations

This study had some limitations which should be taken into consideration. There was only a small sample size and single-center experience (Santa Casa de Curitiba Centre for Heart and Vascular Care). Additionally, the cross-sectional design of our study precludes the establishment of causal or temporal relations between NAFLD and ACS. We included a small number of patients who presented with ACS during a specific period of time, in one hospital. Additionally, the diagnosis of NAFLD was based on ultrasound imaging but was not confirmed by liver biopsy. Upper abdominal ultrasonography has >90% sensitivity and specificity for the detection of liver steatosis. Its sensitivity is significantly lower in those with less than 33% of fatty infiltration compared to liver biopsy or magnetic resonance imaging. This could have resulted in misclassification bias where subjects with mild NAFLD could be classified as normal. This could have resulted in underestimation of the association between NAFLD and CVD. Moreover, ultrasonography cannot distinguish between steatosis alone and NASH, or the stage of the degree of liver fibrosis. Conversely, liver ultrasound is the most widely used noninvasive technique to detect fatty infiltration of the liver in clinical practice, and it has a good sensitivity and specificity in detecting moderate and severe steatosis. A significant proportion of our patients had other traditional risk factors for CVD, but we do not adjust for these important covariates as could be done by a multivariate regression analysis. The lack of a multivariate regression analysis is an important limitation of this analysis.

## 5. Conclusions

In conclusion, our findings suggest that NAFLD is very common in patients with acute coronary syndromes, and the severity of NAFLD detected by ultrasonography is strongly associated with the severity of coronary arteries obstruction. Moreover, the prognostic value of NAFLD in ACS severity stratification remains debatable and will require further prospective studies to confirm the reproducibility of our results.

## Figures and Tables

**Table 1:** Comparison of clinical, laboratory, and ultrasonographic characteristics between patients with and without CAD.

	With CAD	Without CAD	*P* ^*∗*^
Age (years)	59 ± 11.62	54.3 ± 10.83	0.093
Males	63.33% (57)	57.69% (15)	0.65
Type two diabetes	32.22% (29)	30.77% (8)	1
Arterial hypertension	45.56% (41)	65.38% (17)	0.118
Current smokers	31.11% (28)	26.92% (7)	0.81
NAFLD	93.45% (57)	6.56% (4)	*P* < 0.001
NAFLD grade I	94.12% (32)	5.88% (2)	*P* < 0.001
NAFLD grade II	89.47% (17)	10.53% (2)	*P* < 0.001
NAFLD grade III	100% (8)	0%	*P* < 0.001
Without NAFLD	3.4% (4)	17.91% (21)	*P* < 0.001
AST	75.6 ± 116.46	35.6 ± 28.42	0.07
ALT	46.5 ± 30.04	53.5 ± 38.27	0.372
GGT	55.4 ± 44.13	105.3 ± 147.12	0.145
AF	71.8 ± 30.17	88.2 ± 48.04	0.100
Total bilirubin	0.67 ± 0.32	0.73 ± 0.36	0.311
INR	1.11 ± 0.13	1.13 ± 0.18	0.427
LDL cholesterol	98.3 ± 39.09	94.2 ± 39.63	0.639
HDL cholesterol	37.8 ± 11.69	38.2 ± 9.40	0.886
Triglycerides	189 ± 129.11	203.2 ± 85.13	0.513
Creatinine	0.92 ± 0.34	0.82 ± 0.29	0.172

^*∗*^Student *t*-test/Mann–Whitney test.

**Table 2:** Correlation between the presence of NAFLD and CAD.

CAD	NAFLD	
	Present	Absent
Absent	6.58% (5)	43.55% (27)
Present	93.42% (72)	56.45% (35)

## Data Availability

The database will be available from the corresponding author upon request.
